# Preparative supercritical fluid chromatography for lipid class fractionation—a novel strategy in high-resolution mass spectrometry based lipidomics

**DOI:** 10.1007/s00216-020-02463-5

**Published:** 2020-03-04

**Authors:** Harald Schoeny, Evelyn Rampler, Gerrit Hermann, Ulrike Grienke, Judith M. Rollinger, Gunda Koellensperger

**Affiliations:** 1 Department of Analytical Chemistry, Faculty of Chemistry, University of Vienna, Waehringer Strasse 38, 1090 Vienna, Austria; 2 Vienna Metabolomics Center (VIME), University of Vienna, Althanstrasse 14, 1090 Vienna, Austria; 3 Chemistry Meets Microbiology, Althanstrasse 14, 1090 Vienna, Austria; 4 ISOtopic Solutions, Waehringer Strasse 38, 1090 Vienna, Austria; 5 Department of Pharmacognosy, Faculty of Life Science, University of Vienna, Althanstrasse 14, 1090 Vienna, Austria

**Keywords:** Lipidomics, Lipid fractionation, Preparative supercritical fluid chromatography, *Pichia pastoris*, Human plasma, SRM 1950

## Abstract

In this work, a lipidomics workflow based on offline semi-preparative lipid class-specific fractionation by supercritical fluid chromatography (SFC) followed by high-resolution mass spectrometry was introduced. The powerful SFC approach offered separation of a wide polarity range for lipids, enabled enrichment (up to 3 orders of magnitude) of lipids, selective fractionation of 14 lipid classes/subclasses, and increased dynamic range enabling in-depth characterization. A significantly increased coverage of low abundant lipids improving lipid identification by numbers and degree (species and molecular level) was obtained in *Pichia pastoris* when comparing high-resolution mass spectrometry based lipidomics with and without prior fractionation. Proof-of-principle experiments using a standard reference material (SRM 1950, NIST) for human plasma showed that the proposed strategy enabled quantitative lipidomics. Indeed, for 70 lipids, the consensus values available for this sample could be met. Thus, the novel workflow is ideally suited for lipid class-specific purification/isolation from milligram amounts of sample while not compromising on omics type of analysis (identification and quantification). Finally, compared with established fractionation/pre-concentration approaches, semi-preparative SFC is superior in terms of versatility, as it involved only volatile modifiers and salt additives facilitating any follow-up use such as qualitative or quantitate analysis or further purification down to the single lipid species level.

**Electronic supplementary material:**

The online version of this article (10.1007/s00216-020-02463-5) contains supplementary material, which is available to authorized users.

**Figure:**
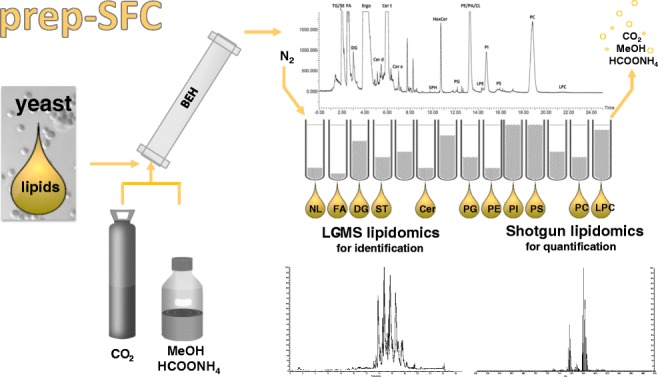
Graphical Abstract

## Introduction

High-resolution mass spectrometry (HRMS) has evolved as key technology in the field of lipidomics showing an unrivaled potential to pursue quantification and lipid species identification in parallel [1]. Shotgun lipidomics, i.e., direct infusion HRMS (DI-HRMS) [2] enables accurate quantification of hundreds of lipids in complex samples. Evidently, the combination of HRMS and liquid chromatography (LC) is powerful when aiming at in-depth characterization of the lipidome, as increased dynamic range improves the lipidome coverage, which in turn enables higher numbers of identified species within one analytical run [3, 4]. In this work, for the first time, both HRMS and LC-HRMS have been combined to lipid class-specific fractionation as obtained by upscaling analytical supercritical fluid chromatography (SFC). The aim has been to create a workflow enabling purification/fractionation of as many lipid classes as possible at semi-preparative scale together with in-depth characterization of the lipidome. At analytical scale, SFC-based lipid analysis has been introduced in the late 1980s [5–7] but the number of studies had remained low, until significant technological improvements (e.g., the introduction as sub 2 μm particles, backpressure regulation and improved injection systems) have led to a renaissance of SFC in lipidomics [8]. Above all, the facilitated combination with mass spectrometry (MS) accelerated these developments. Bamba et al. [9] has pioneered the field. Studying different column chemistries, separation of phospholipids, glycolipids, neutral lipids, and sphingolipids in relatively short time (15 min) could be accomplished. Depending on stationary phase selection, SFC separation is either governed by head group or by fatty acid chain length, degree of saturation and double bond position. A reversed-phase-like separation has been proposed when using non-polar stationary phases [10–13], while HILIC type of separation is enabled when using polar stationary phases. The current state-of-the-art method has been introduced by Lísa et al. [14] who have developed a powerful high-throughput SFC high-resolution mass spectrometry (HRMS) method to separate non-polar and polar lipids within one run. More specifically, by ultra-high performance SFC (UHPSFC) on an ethylene-bridged hybrid stationary phase with 1.7 μm particles, a separation of 30 lipid classes has been achieved. The optimized chromatographic gradient starting with pure non-polar CO_2_, which is then followed by the addition of up to 51% methanol/water (99:1, v/v) containing 30 mM of ammonium acetate, is key to separate both polar and non-polar lipids. Further applications of analytical SFC in lipidomics are comprehensively summarized elsewhere [15–18]. However, the lipid class-specific SFC separation introduced by Lísa et al. remains unrivaled in both separation speed and coverage up to date.

Based on the SFC studies on analytical scale, in this work, we have addressed the development of a short, semi-preparative SFC method for both polar and non-polar lipids within one run to separate milligram amounts of dry lipid extract. Most well-established preparative lipid separations resort to techniques developed already decades ago, such as thin layer chromatography (TLC) [19, 20], column chromatography (CC) [21, 22], preparative high-performance liquid chromatography (prep-HPLC) [23, 24], or solid-phase extraction (SPE) [25–28]. As a major drawback, not all of them allow for lipid class fractionation. On top of that, the techniques, regardless whether implemented offline or online, are time and solvent consuming, often requiring multiple steps. For example, a combined SPE protocol addressing fractionation of 11 different lipid classes [26, 28] has implied the use of 12 different solvent mixtures, two cartridges per sample, and several hours of subsequent drying steps. While preparative SFC (prep-SFC) is widely used in pharmaceutical sciences [29, 30], its application is less common in the realm of lipids and rather focused on purification of selected classes [20, 21] (e.g., non-polar lipid classes [31–34], polyunsaturated fatty acids (PUFAs) [35], or phospholipids (PL) [31]). A comprehensive fractionation attempt covering the whole lipidome is still lacking. However, due to the key advances of (1) easy removal of supercritical CO_2_ by evaporation upon fractionation, (2) avoidance of complex buffer systems, which complicate the further use of the obtained fractions, and (3) relatively short run time compared with other chromatographic mechanisms, prep-SFC shows great potential for a global lipidome fractionation.

The novel workflow has been applied to the lipidome characterization of *Pichia pastoris* (Guillierm.) Phaff 1956 (*Komagataella phaffii* Kurtzman) [36]. The yeast strain *Pichia pastoris* is a well-known cell factory related to lipidomics but not as well studied as the yeast *Saccharomyces cerevisiae* [37–39]. The existing lipid studies have served as a reference [40–43] for this work.

## Materials and methods

**Fig. 1:**
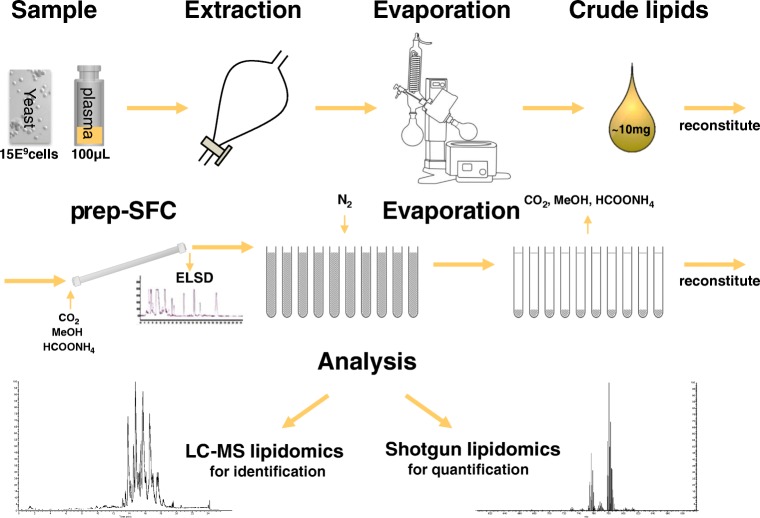
Overview of the applied workflow including sample preparation, SFC separation, and MS analysis

In Fig. 1, a simplified workflow is shown. The different working steps are described below and in the Electronic Supplementary Material (ESM).

### Sample preparation

A detailed sample preparation procedure can be found in the ESM. Briefly, the yeast was fermented according to a previously established protocol [44] and the cells were extracted following the procedure of Folch [45]. Human plasma was purchased from the National Institute of Standards and Technology (NIST), USA, as standard reference material 1950 (SRM 1950) and also extracted to an adopted Folch extraction.

### Semi-preparative supercritical fluid chromatography for lipid class fractionation

For semi-preparative SFC, the Waters Prep-15 SFC system was used. The system comprised a fluid delivery module (connected to an Accel 500 LC chiller by Thermo Fisher Scientific), a Waters 2767 sample manager, a ten-port column oven, a back pressure regulator, a heat exchanger, a make-up pump, a Waters 2998 Photo Diode Array (PDA, at 210 nm), and a Waters 2424 Evaporative Light Scattering Detector (ELSD). The software MassLynx V4.1 was used for controlling the chromatography.

A Viridis BEH column (250 × 10 mm, 5 μm, Waters Corporation) was used at 60 °C column temperature. The following gradient was used with pure CO_2_ as solvent A and methanol (MeOH) with 30 mM ammonium formate (1.892 g dissolved prior in 5 mL water for 1 L MeOH) as modifier (solvent B): 0–4.0 min 5% B, 4.0–17.0 min ramp to 55% B, 17.0–25.0 min 55% B, 25.0–25.5 min ramp down to 5% B, and 25.5–27.0 min 5% B as equilibration step.

The injection volume was 300 μL and the injector needle was washed with methanol for 5 s prior to each injection. Samples were dissolved in chloroform. The flow rate was 15 mL min^−1^, the backpressure was set to 120 bar, and after the column, the eluents were combined with a methanol make-up flow of 3 mL min^−1^ to facilitate the detection and the fraction collection. The fractionation time events can be found in the ESM (Table S1). The retention time stability was checked each time prior fraction collection. The fractions were collected, dried under nitrogen, and stored at − 80 °C prior analysis.

### High-resolution mass spectrometry shotgun lipidomics

For the direct infusion analysis of the fractions, a robotic nanoflow ion source TriVersa NanoMate (Advion BioSciences, Ithaca, NY, USA) was coupled to a high-field Q Exactive HF™ quadrupole-Orbitrap mass spectrometer (Thermo Fisher Scientific, Bremen, Germany). The dried samples were diluted in isopropanol (IPA)/MeOH/CHCl_3_ 4:2:1 (v/v/v) containing 7.5 mM ammonium formate and 30 μL was placed in a 96-well twin.tec® plate (Eppendorf, Hamburg, Germany). Nano-electrospray ionization (nano-ESI) chips with spraying nozzles of 5 μm nominal internal diameter were used and the whole NanoMate was controlled by the Chipsoft 8.3.1 software (both Advion BioSciences). The following settings were applied: ionization voltage 1.25 kV(+)/− 1.25 kV (−); backpressure 0.9 psi; capillary temperature 250 °C; S-Lens radio frequency (RF) level 50.

Each sample was measured for 17 min and polarity switching was triggered after 8 min (afterwards 1 min for equilibration) via contact closure signal by the mass spectrometer as described previously [46]. For each polarity, only MS1 spectra were acquired at the beginning for 30 s before 200 data independent acquisition (DIA) scans alternated with a MS1 scan for quantification. In MS1, the resolution was set to 240,000, the automatic gain control (AGC) target to 1e6, and the maximum injection time (IT) to 150 ms. For the DIA scans, a resolution of 60,000 was applied and the AGC target and the max IT was set to 2e5 and 130 ms, respectively. Normalized collision energy (NCE) of 25 was used in positive mode and 28 in negative mode. The scan range was set to *m*/*z* 200–1600 in both modes.

Data evaluation for identification and quantification was performed with LipidXplorer 1.2.7 software [47]. The spectra were imported into a Master Scan database using the following settings: mass tolerance 5 ppm, min.; occupation of 0; intensity threshold 10,000 (MS1)/5000 (MS2); resolution 260,000 (MS1)/65,000 (MS2); resolution gradient − 102 (MS1)/− 60 (MS2). The molecular fragmentation query language (MFQL) files used for identification can be found in the ESM. Limits of quantification (LOQ) were calculated according to EURACHEM, The Fitness for Purpose of Analytical Methods, 2nd edition (2014), by repetitive injections of a low concentrated internal standard.

### Reversed-phase chromatography high-resolution mass spectrometry

For reversed-phase (RP) chromatography of lipids, an Acquity HSS T3 (2.1 mm × 150 mm, 1.8 μm, Waters) with a VanGuard Pre-column (2.1 × 5 mm, 100 Å, 1.8 μm) was used. The column temperature was set to 40 °C and the flow rate to 250 μL min^−1^. Acetonitrile (ACN)/H_2_O (3:2, v/v) was used as solvent A and IPA/ACN (9:1, v/v) as solvent B, both containing 0.1% formic acid and 10 mM ammonium formate. The following gradient was applied: 0–2.0 min 30% B, 2.0–17.0 min ramp to 75% B, 15.0–17.0 min ramp to 100% B, 17.0–22.0 min 100% B, 22.0 min fast switch to 30% B, and equilibrated at the starting conditions for 5 min (22.0–27.0 min 30% B). The injector needle was washed with 75% isopropanol, 25% H_2_O, and 0.1% formic acid for 5 s prior to each injection. The temperature of the autosampler was set to 10 °C. The same samples as for shotgun lipidomics were used (IPA/MeOH/CHCl_3_ 4:2:1 (v/v/v) containing 7.5 mM ammonium formate) and the injection volume was 2 μL. A high-field Thermo Scientific™ Q Exactive HF™ quadrupole-Orbitrap mass spectrometer equipped with an electrospray source was used for HRMS. The ESI source parameters were the following: sheath gas 35, auxiliary gas 5, spray voltage 2.8 kV in negative and 3.5 kV in positive mode, capillary temperature 220 °C, S-Lens RF level 30, and auxiliary gas heater 300 °C. Spectral data were acquired in profile mode.

The full MS runs in positive mode were acquired in the range of *m/z* 200–2000 at a resolution of 120,000, an AGC target at 1e6, and a maximum IT of 200 ms. Data-dependent MS2 (ddMS2) fragmentation spectra were acquired for identification in positive and negative mode. For both, a Top8 method with a NCE of 25(+)/28(−) and an isolation window of *m/z* 1 was applied. The resolution in the MS2 was set to 30,000, the AGC target to 2e5 (minimum 8e3), and the max IT to 60 ms. The dynamic exclusion of triggered *m/z* was set to 15 s. Both an inclusion and an exclusion list were used for the possible lipids in yeast and the background compounds identified in a blank run, respectively.

Lipid identification was performed with Lipid Data Analyzer (LDA) 2.6 [48] and LipidSearch 4.2 from Thermo Scientific, in which following filters were applied: RT tolerance 0.25 min, m-score threshold 5, ID quality filter A,B,C (D- only for free fatty acids and cardiolipins), calculate unassigned peak area TRUE, and top rank filter TRUE. The identifications were curated manually following the criteria in Table S2 (see ESM).

## Results and discussion

### Semi-preparative supercritical fluid chromatography method development

The developed semi-preparative class-specific separation was based on the work of Lísa et al. [14]. This rigorously optimized analytical SFC utilizing sub 2 μm particle stationary phases enabled the separation of polar and non-polar lipid classes within 6 min. Accordingly, the selection of column chemistry involving ethylene-bridged hybrid (BEH) material and the optimized parameter (such as column temperature, water-additive composition, modifier gradient) served as a starting point of the upscaling process. As a drawback, the 400 bar pressure limit of the prep-SFC system (suggested operating range, 100–200 bar) only permitted the use of stationary phases with 5 μm particle size instead of sub 2 μm. A 10-mm column operated at flow rates of 15 mL min^−1^ was implemented. Additionally, the different injection modes between prep-SFC and analytical SFC needed to be considered. Indeed, the former system provides a so-called modifier-stream injection mode, in which the sample is injected into the modifier (solvent B) before it is mixed with CO_2_. This mode benefits from reduced impact of the injection solvent compared with a mixed-stream injection [49, 50] established in analytical SFC. However, as the modifier-stream injection mode only works while using a modifier, a minimum of 5% modifier is required. The method developed by Lísa et al. [14] involved 100% CO_2_ (solvent A) as starting condition and column temperatures of 60 °C, a prerequisite for the achieved excellent separation of non-polar lipids. The starting conditions of 100% CO_2_ (A) could not be accomplished by the applied prep-SFC system (Waters Prep-15 SFC). Hence, the separation power regarding non-polar lipid classes was compromised. On the other hand, using the modifier-stream injection mode, the impact of sample matrix on the separation power was reduced. The final SFC method was based on a CO_2_ (A) methanol-ammonium formate (B) gradient. The addition of volatile salts such as ammonium formate to the modifier was crucial with regard to peak shape of polar lipids. Omitting the salt additives was reported to hamper class-specific separation for these lipids. As a major advantage, the follow-up characterization or simply the use of the lipid class fractions obtained by prep-SFC was straightforward requiring no additional sample preparation steps as only volatile modifiers and salt additives were involved. Compared with other preparative lipid fractionation methods such as TLC, CC, prep-HPLC, and SPE, prep-SFC is fast, cost-efficient, less laborious, environmentally friendly [51], and has additional upscaling potential. In this work, up to 300 μL volumes could be injected containing 10 mg mL^−1^ dry lipid extract, which is 3 mg dry lipid extract compared with 0.5 μg lipid dry mass sample intake in analytical SFC (0.5 mg mL^−1^, 1 μL) [14].

**Fig. 2:**
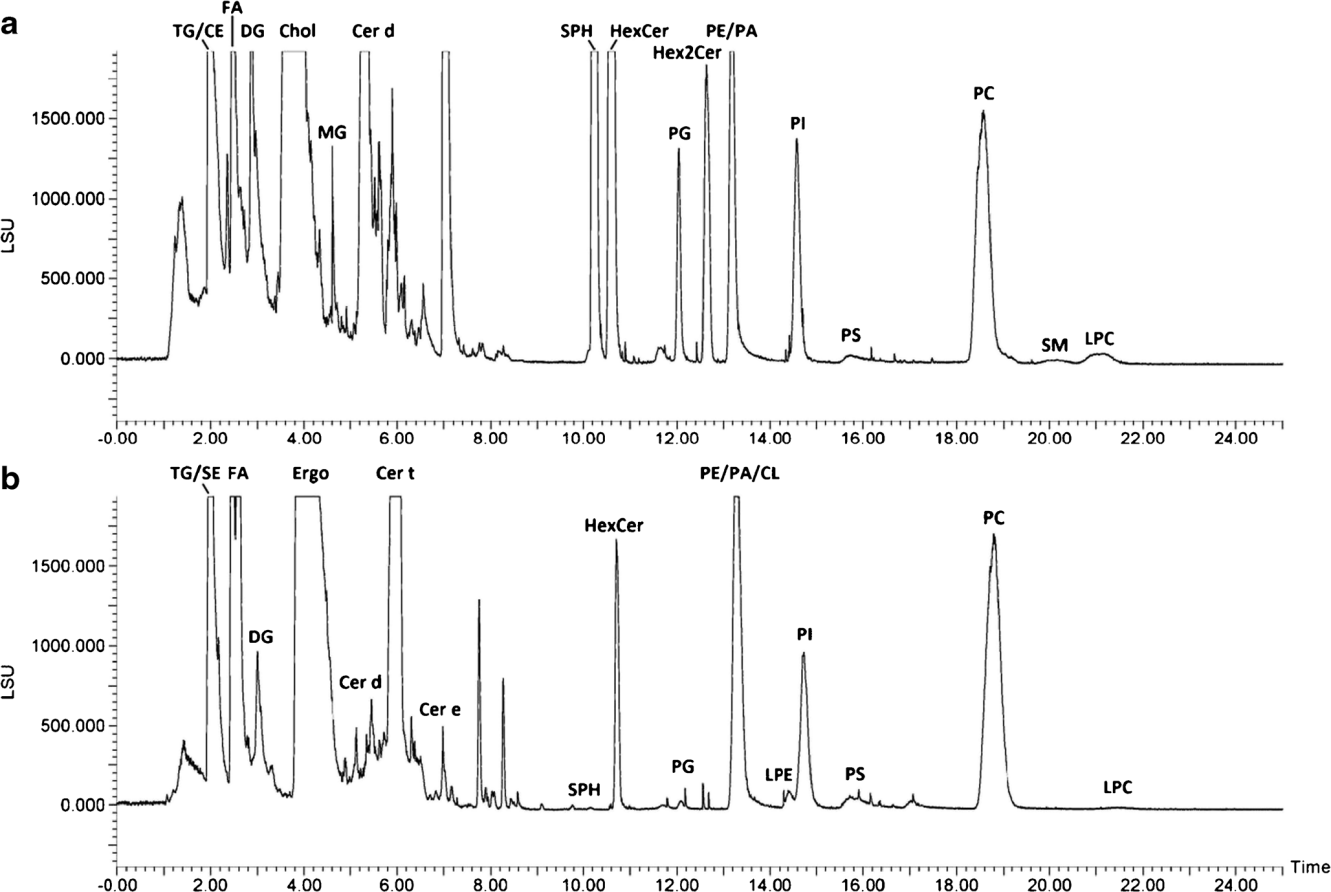
Supercritical fluid chromatogram of a **a** multi-lipid mix and **b** yeast (*Pichia pastoris*) detected with ELSD. Peak annotation: TG, triacylglycerols; CE, cholesteryl esters; SE, steryl esters; FA, fatty acids; DG, diacylglycerols; Chol, cholesterol; Ergo, ergosterol; MG, monoacylglycerols; Cer, ceramides (d,t,e, di,tri,tetra hydroxylated); SPH, sphingosine bases, HexCer, hexosyl ceramides; PG, phosphatidylglycerols; Hex2Cer, dihexosyl ceramides; PE, phosphatidylethanolamines; PA, phosphatidic acids; CL, cardiolipins; PI, phosphatidylinositols; LPE, lysophosphatidylethanolamines; PS, phosphatidylserines; PC, phosphatidylcholines; SM, sphingomyelins; LPC, lysophosphatidylcholines. “/” indicates co-eluting lipid classes

Figure 2 shows the optimized prep-SFC separation monitored by evaporative light scattering detector (ELSD) for lipid standards and yeast samples. As can be readily observed, a broad lipid polarity range was covered (from non-polar lipids such as triacylglycerols (TG) to polar lipids such as lysophosphatidylcholines (LPC)). In analogy to analytical SFC, neutral lipids showed lower retention compared with polar lipids. The elution orders were also comparable, with the exception of the lipid classes phosphatidylserines (PS), phosphatidic acids (PA), and cardiolipins (CL). The final prep-SFC method featured a separation of 22 fractions within a runtime of 27 min. The collection of the lipid fractions was performed after the backpressure regulator, which was responsible of the density control along the column to maintain the solubility and the retention behavior of the analytes. Additional building blocks (heater, gas-liquid separator, make-up solvent) improved the recovery of the eluting fractions. Pure methanol was used as make-up solvent and the lipids were collected in defined time windows. First, the fraction collection timing was based on the detection of non-volatile compounds by ELSD only. This timing was fine-tuned by an iterative approach involving offline HRMS-based lipidomics screening of the fractions. The exact timing is given in Table S1 (see ESM).

**Fig. 3:**
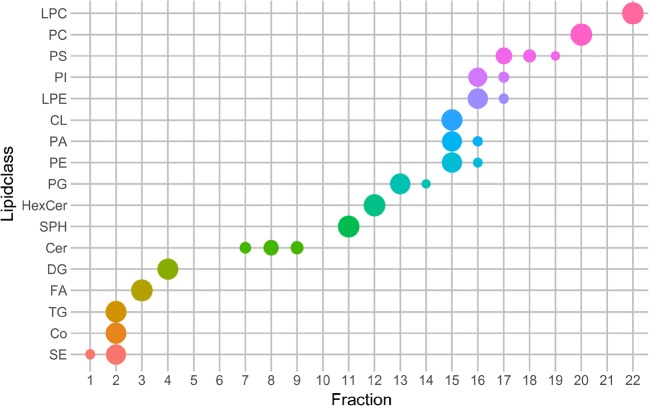
Distribution of each yeast lipid class over the fractionated SFC run. Values are calculated by the summarized area values of the lipid species of each lipid class obtained by LipidSearch. The size of the points accounts for the relative area of each lipid class over all fractions (smaller points indicate a distribution over several fractions). Colors emphasize the different lipid classes on the y-axis

Using standards and yeast samples, it could be shown that lipid class-specific fractionation was successfully accomplished for 14 different lipid classes or subclasses (FA, DG, ST, MG, SPH, HexCer, PG, Hex2Cer, PC, SM, LPC, Cer d, Cer t, Cer e) paving the way to further purification, in-depth profiling, or facilitating a detailed fatty acyl chain composition determination via gas chromatography-mass spectrometry (GC-MS) as previously addressed by TLC or SPE [52, 53]. Figure 3 gives a detailed overview of the fractionated lipid classes in yeast; the retention time of cholesterol, MG, Hex2Cer, AcCa, and SM was determined by the multi-lipid mix only. Early eluting non-polar lipid classes such as TG, SE, and ubiquinones (coenzymes, Co) could not be separated due to the compulsory minimum amount of modifier upon injection. Additionally, class-specific fractionation was only partly successful for PLs which could be to some extent attributed to peak tailing. While PG, PC, and LPC were clearly separated, others were only partially fractionated (e.g., fraction 15 contained PE/PA/CL; fraction 16 contained PI/LPE).

However, the fractionation strategy allowed overcoming several selectivity challenges of MS analysis, e.g., isomeric classes PC and PE (also LPC and LPE) were separated. PC, SM, and LPC, all producing the head group fragment *m/z* 184 in positive mode due to their choline-containing head group, were separated. Finally, important classes showing corresponding in-source fragments or degradation products were successfully fractionated into different lipid classes such as sphingolipids (SPH, Cer, HexCer, and Hex2Cer) sterol/sterol esters or lysophospholipids and their corresponding phospholipids.

**Fig. 4:**
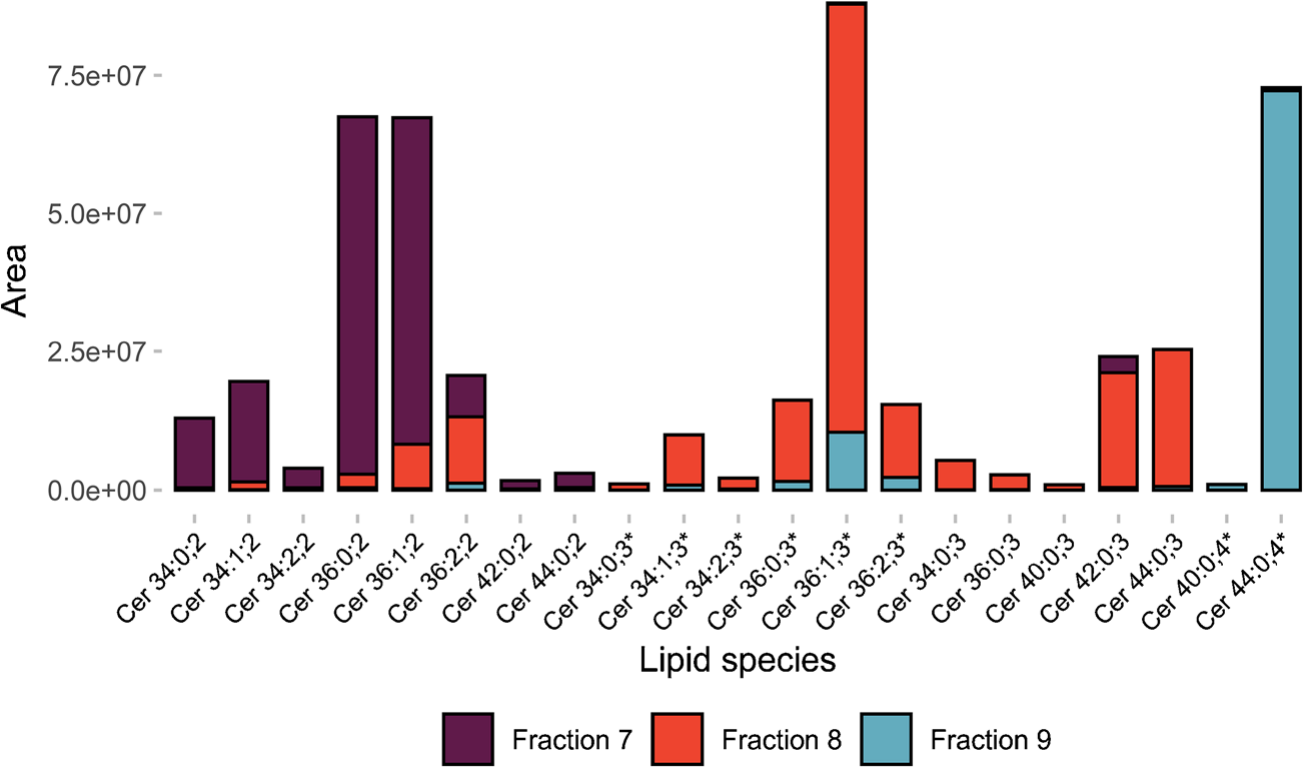
Distribution of ceramides in the ceramide-containing fractions. Ceramides ordered after the number of hydroxyl groups in the three fractions, where ceramides were identified (fraction 7–9). Lipids marked with asterisk have a hydroxyl group on the fatty amid chain

Interestingly, for ceramides, differences in hydrophilicity due to long-chain bases (LCB) as well as hydroxylated and non-hydroxylated fatty amid chains [54] led to SFC separation within the class. Using *P. pastoris*, ceramides were recovered in three fractions (7–9). Ceramides containing two hydroxyl groups eluted in a separate fraction as well as ceramides with three respectively four hydroxyl groups (see Fig. 4).

### Identification, purification, and quantification of lipids from *Pichia pastoris*

**Fig. 5:**
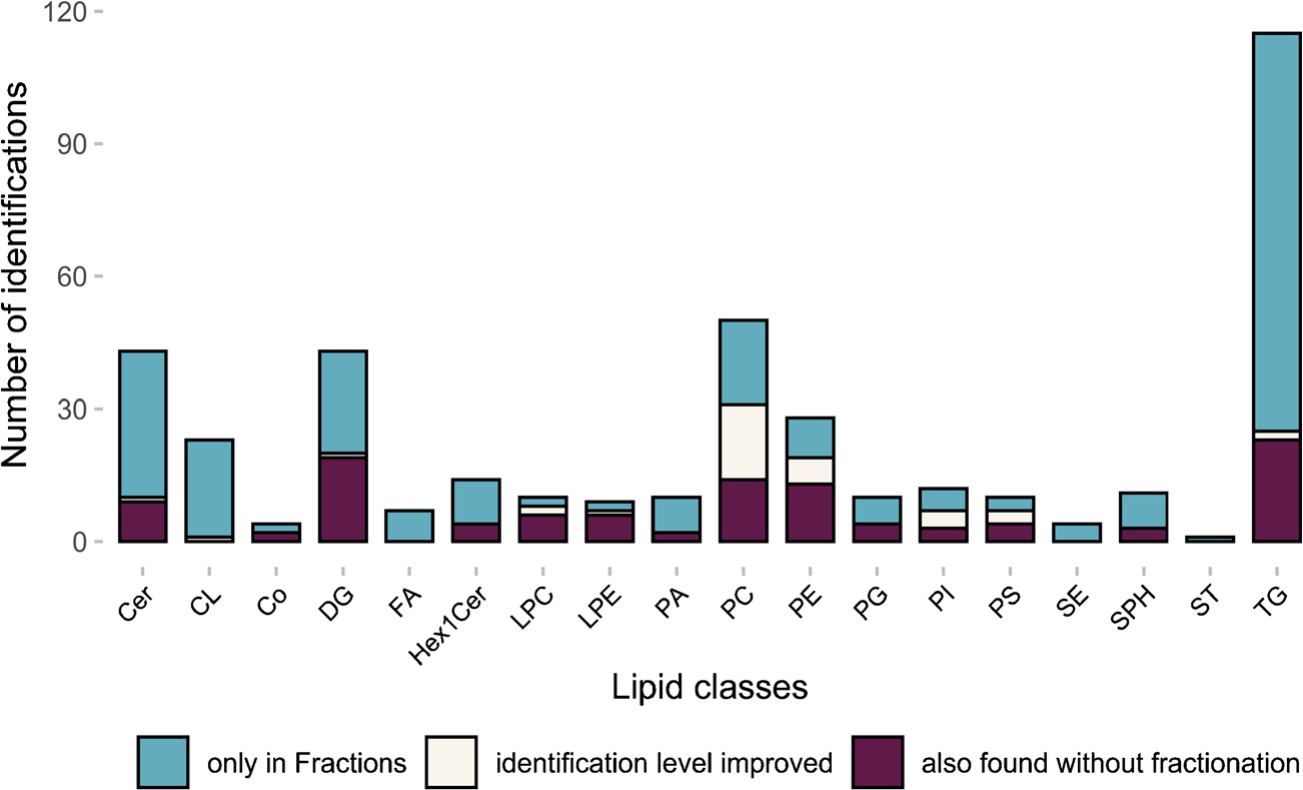
Improved lipid identification in yeast with SFC fractionation. The total number of identifications over all fractions of the fractionated yeast (whole bar) compared with identifications in the non-fractionated full extract (purple and white bar) was more than doubled (404 compared with 150). The white bar corresponds to the lipids identified in both fractionated and non-fractionated full extract, but the level of identification was improved in the fractionated yeast (molecular species level, e.g., PC 16:0_16:1 instead of lipid species level, e.g., PC 32:1). The blue bar shows the number of lipids only identified in the fractionated yeast

In a next step, the workflow was evaluated for in-depth characterization of a yeast lipidome. *Pichia pastoris* selected as comprehensive lipidomics is still behind [40–43], when compared with the well-known yeast *Saccharomyces cerevisiae.* The collected SFC fractions were analyzed by RP-LC-HRMS using gradient elution of 22 min for identification and shotgun HRMS for quantification (see supplementary Excel table “Yeast_quant_shotgun_results” in the ESM). In parallel, non-fractionated yeast extracts were analyzed by RP-LC-HRMS to compare the number of identifications. Overall, a tremendous increase (by 170%) of lipid identification was achieved by the novel workflow (see Fig. 5). In total, 404 lipid species of 18 different lipid classes from six different lipid categories were identified either on the lipid or molecular species level in the fractions (corresponds to the whole bar per class). This number exceeded common workflows such as shotgun lipidomics (250 in *S. cer.* [38]) and HPLC analysis (ca. 200 in *P. pastoris* [40, 55]) Without SFC prefractionation, only 150 lipids (purple and white bar) were identified in *Pichia pastoris* using RP-HRMS in this work. The novel strategy proved to be advantageous in the case of low abundant lipid classes (Cer, FA, SPH) and lipids with low ionization efficiency (SE, ST). Moreover, due to the reduced complexity, the number of PLs, both in the isolated classes, e.g., PC and PG, as well as in the mixed fractions, e.g., PE/PA/CL, was increased. Finally, not only the number of identified lipids could be enhanced, also the degree of identification was improved (blue bar). An example is the lipid class PC. This class ionize highly efficiently in positive mode but the corresponding MS2 spectra by higher-energy collisional dissociation (HCD) only delivers head group-specific fragments that cannot help to identify the fatty acyl chain composition (compare lipid species level (e.g., PC 32:1) and molecular species level (e.g., PC 16:0_16:1)). However, in negative mode, the main fragments are the fatty acyl chain fragments, which make this mode preferable for identification. Unfortunately, in this mode, the ionization efficiency is less and higher concentrations, as obtained with SFC fractionation, can double the number of identified lipids on the molecular species level (see Fig. 5, also for PE, PS, PI). A detailed list of identified lipids (including identification level) is given in the supplementary Excel table (see ESM). The list was obtained after thorough corroboration of first screening data obtained by the application of commercial software tools (LipidSearch 4.2 from Thermo Scientific). Orthogonal open-source software tools based on fragmentation rules (Lipid Data analyzer 2.6 [48] and LipidXplorer 1.2.7 [47] for LC and shotgun data, respectively) were used to confirm the identifications based on in silico-generated database search. As a drawback, this strategy is restricted to classes, where reliable fragmentation rules are available. Therefore, for some lipids, the validation of lipid identification resorted to a probability check through literature data [37, 38, 40–43]. Only a limited number of lipids were confirmed by authentic standards. It has to be mentioned that following the definition according to Schymanski et al. [56], only a standard matched identification corresponds to level 1 identification. Thus, all other lipids were identified on level 3 as the exact structure (position of double bonds, stereochemistry) cannot be determined with the applied HCD MS2 fragmentation. The total list can be found in the supplementary Excel tab “Yeast_ident_LC” (see ESM). Overall, 317 out of 404 lipid species could be validated by orthogonal approaches.

### Validation of prep-SFC for offline quantification

In a next step, the SFC fractionation was combined to shotgun HRMS and was validated with regard to quantification accuracy. Proof-of-principle experiments were performed using the established internal standardization strategy (one-point calibration with one deuterated internal standard per lipid class) and a standard reference material from NIST, USA (SRM 1950), of human plasma. A recent international interlaboratory comparison provided consensus values for a large number of lipids [57]. An online tool denoted as LipidQC [58] facilitated cross-validation. The concentration was calculated according the following equation:

$$ {\mathrm{conc}}_{\mathrm{Analyte}}\ \left[\upmu \mathrm{mol}\ {\mathrm{L}}^{-1}\right]={\mathrm{conc}}_{\mathrm{ISTD}}\cdotp \frac{{\mathrm{Intensity}}_{\mathrm{Analyte}}}{{\mathrm{Intensity}}_{\mathrm{ISTD}}} $$

**Fig. 6:**
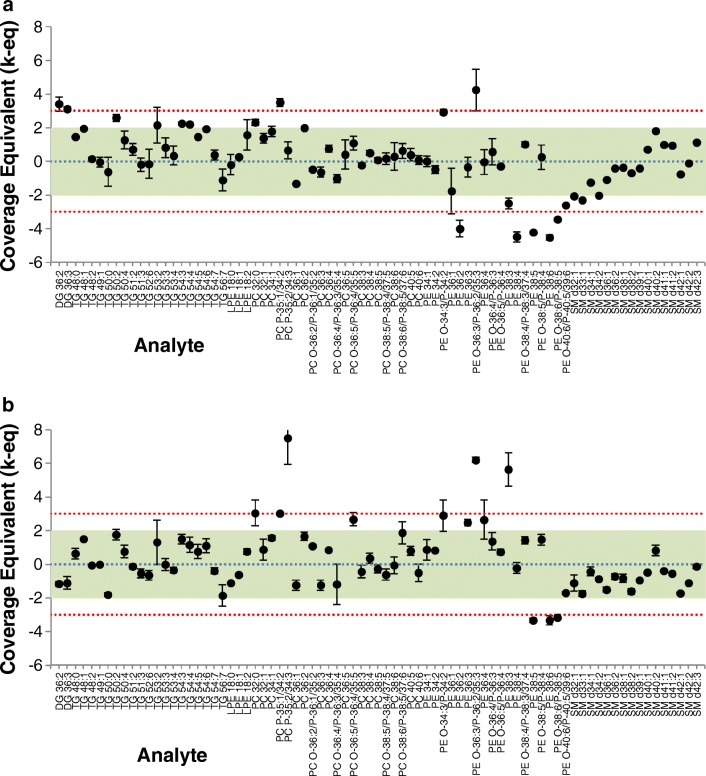
Accuracy assessment for SRM 1950 - “Metabolites in Frozen Human Plasma.” Values are presented as normalized coverage equivalents at the mean (dots) and stdev (error bars, *N* = 2) of measurements, overlaid onto the consensus mean value (blue line) and uncertainty (95% coverage, green region; 99% coverage, red region). **a** Fractionated with SFC. **b** Non-fractionated full extract. See ESM Tables S3 and S4 for detailed results. Graphic is produced with LipidQC [58]

As the internal standard was added prior extraction and therefore also before SFC separation, any losses during the process influence the internal standard and the analytes equally. Even a lower recovery rate can still lead to a decreased complexity in the following analysis, thus enabling quantification of low abundant lipid species. Figure 6 gives an overview of the accuracy assessment for both fractionated and non-fractionated plasma. The figure compares quantitative values (> LOQ by both approaches) for the lipid classes DG, TG, LPE, PC, PE, and SM. Overall, 70 out of 79 lipids were absolutely quantified within the 99% confidence interval by both approaches, which in turn confirmed the quantitative capability of SFC fractionation at semi-preparative scale. Finally, the quantitative semi-preparative SFC/shotgun lipidomics workflow was applied to *P. pastoris*. The results of this study are summarized in the supplementary Excel table (see ESM). It can be readily seen that starting from 1 g yeast resulting in 10 mg/mL lipid extract following sample preparation, high microgram amounts of lipid classes can be obtained.

## Conclusions

The presented novel lipidomics workflow proved to be very versatile. The key advantages can be summarized as follows: (1) The class-specific fractionation by SFC offered reduced complexity and enrichment of low abundant lipids and lipid classes, respectively, thus overall enhancing the number of identified lipids (compare shotgun lipidomics (250 in *S. cer.* [38]) and HPLC analysis (ca. 200 in *P. pastoris* [40, 55]); (2) the workflow enabled absolute quantification as shown in a validation study using SRM1950; and (3) SFC commonly accepted as a green method provided “ready-to-use” lipid class fractions. Thus, follow-up lipid analysis is not limited to LC-MS-based assays. Any other additional lipid characterization method can be applied such as GC-MS analysis following hydrolysis and methylation (fatty acid methyl ester (FAME) analysis to determine the fatty acyl chain composition of each class) or structural elucidation via NMR. Additionally, in the future, isotopically labeled and non-labeled lipid standards/fractions could be produced from any organism of interest. The relatively short run time of 27 min, the automatization, and the use of CO_2_ makes this fractionation method attractive for many applications.

## Electronic supplementary material

ESM 1 (PDF 949 kb)

ESM 2 (XLSX 2.44 mb)
