# Supplementary material for: Preparative supercritical fluid chromatography for lipid class fractionation—a novel strategy in high-resolution mass spectrometry based lipidomics
Source: Anal Bioanal Chem. 2020 Mar 4;412(10):2365–74. doi: 10.1007/s00216-020-02463-5 (PMC7118041; doi:10.1007/s00216-020-02463-5)
Supplement: Supplementary file 1 — (PDF 949 kb) [file 216_2020_2463_MOESM1_ESM.pdf]

Analytical and Bioanalytical Chemistry

Electronic Supplementary Material

Preparative supercritical fluid chromatography for lipid class fractionation – a novel strategy in high-resolution mass spectrometry based lipidomics

Harald Schoeny, Evelyn Rampler, Gerrit Hermann, Ulrike Grienke, Judith M. Rollinger, Gunda Koellensperger

Additional files available under 10.1007/s00216-020-02463-5

Standards and solvents

Acetonitrile (ACN), isopropanol (IPA), methanol (MeOH), chloroform ($CHCl_3$) and water were of LC-MS grade and ordered at Fisher Scientific (Vienna, Austria) or Sigma Aldrich (Vienna, Austria). Ammonium formate was ordered as eluent additive for LC-MS at Sigma Aldrich. Formic acid was also of LC-MS grade and ordered at VWR International (Vienna, Austria).

Lipid standards were purchased from Avanti Polar Lipids, Inc. (Alabama, USA). All standards were weighed and dissolved in an appropriate solvent. A multi-lipid mix was prepared in the used sample solvent. SPLASH® Lipidomix® Mass Spec Standard was purchased from Avanti Polar Lipids, Inc. (Alabama, USA).

Fermentation of *Pichia pastoris*

Yeast fermentation was conducted according to a previously established protocol [1]. Shortly, a cell culture of *Pichia pastoris* was grown on glucose as the only carbon source. The whole lipidome of the yeast can be uniformly ^{13}C labeled by using $U^{13}C$-glucose, whereas the labeling efficiency was improved by cultivating yeast precultures in shaker flasks prior to the actual inoculation of the fermenter. A BioFlo 310 Fermenter (New Brunswick™) was used to perform the fed-batch fermentation for 72 h. The fermentation broth was transferred into 50 mL falcon tubes and centrifuged for 5 min at 4000 rcf at 4 °C. The supernatant was discarded and the cell pellet was stored at −80 °C until further processing.

Lipid extraction of yeast cells

The extraction followed the procedure of Folch [2]. A dried cell suspension of *Pichia pastoris* (1 g ±0.2 g equals to 1.5 E10 cells) was washed with 20 mL ammonium bicarbonate (ABC) buffer (150 mM) to remove salts from the matrix. It was centrifuged for 10 min at 1000 rcf and the supernatant was descanted and discarded. The pellet was again dissolved in 15 mL ABC buffer and mixed vigorously. During all steps the sample was stored at ice if possible.

The cell suspension was aliquoted 16 times into 1 mL fractions into a 2 mL polypropylene tube, which is suitable for mechanical disruption device, in which glass beads had already been inserted with an amount corresponding to a liquid equivalent of 400 µL. It had to be aliquoted because the used *Peqlab Minilys* for tissue homogenization was only suitable for this 2 mL tubes. Each tube was shaken three times for 10 s at full speed respectively and between each lysis step the sample was cooled on ice for one minute.

Afterwards approximately 750 µL lysate of each tube was collected in a 1000 mL glass flask with a teflon lid. 500 mL (745 g) of $CHCl_3$ and 250 mL (198 g) methanol were added and the flask was shaken on ice for 1.5 h at 200 rpm. Afterwards it was removed from ice and 250 mL ABC buffer (150 mM) was added and shaken to initiate phase separation. After the two phases were clear the upper phase was put into Falcon tubes by an adjustable 10 mL pipette. The interphase which contained the insoluble proteins was collected separately. After the lower phase was almost totally free of any upper layer, it was put into a 500 mL round bottom flask by a DLAB Levo ME electric pipette with 50 mL glass tips. The organic lower phase was evaporated to dryness in a rotary evaporator, transferred into a 2 mL glass vial with $CHCl_3$, dried again and stored at -20 °C until further treatment. The lipids were dissolved in 1 mL $CHCl_3$ to obtain a concentration of 1.5 billion cells mL^{-1}.

Lipid extraction of human plasma

To a 100 µL plasma sample aliquot (standard reference material 1950, NIST, USA), which was placed on ice in a glass tube with a Teflon-lined cap, 1.253 mL Methanol, 80 µL SPLASH® Lipidomix® Mass Spec standard and 2.667 mL Chloroform were added. After each addition the tube was shaken vigorously. The mixture was shaken (400 rpm) for 1 h at 4 °C (ThermoMixer C, Eppendorf). After that, 1 mL ABC Buffer (150 mM) was added and the phase separation was done at room temperature. The aqueous phase was collected and the organic lower phase was put in a glass vial. The aqueous phase was re-extracted with 667 µL $CHCl_3$ and 333 µL MeOH. The combined phases were dried under nitrogen and stored at -20 °C until SFC measurement. Before analysis, the dried lipids were dissolved in 800 µL $CHCl_3$ and transferred into a 2 mL glass vial.

Table S1 Collection time ranges and fraction number for the SFC run

**Fr.**	**1**	**2**	**3**	**4**	**5**	**6**	**7**	**8**	**9**	**10**	**11**
**t [min]**	1-2	2-2.5	2.5-3	3-3.5	3.5-5	5-6.4	6.4-7.2	7.2-7.9	7.9-8.5	8.5-10	10-10.6
**Fr.**	**12**	**13**	**14**	**15**	**16**	**17**	**18**	**19**	**20**	**21**	**22**
**t [min]**	10.6-11.3	11.3-12.2	12.2-12.9	12.9-14.2	14.2-15.5	15.5-16.8	16.8-17.6	17.6-18.2	18.2-19.2	19.2-20.5	20.5-23

Table S2 LipidSearch filter criteria

Class	Criteria	Class	Criteria
AEA	+H, ethanolamine and M+H frag	LPE	+H, NL, isomer separation→ 2 peaks
Cer	+H, SPH frag, A or B grade	PC	+H, 184 frag
CL	+H/-H/-2H, DG frag in pos, FA in neg	PE	+H/+Na, NL
Co	+NH_4, M-Q frag, alkyl-pattern	PG	+NH_4, NL
DG	+NH_4, NL (FA), alkyl-pattern, A or B grade	PI	+NH_4, NL
FA	-H	PS	+H/-H/+Na, NL in pos, FA in neg
HexCer	+H, SPH frag and NL, A grade	SPH	+H, waterloss
LPC	+H, 184 frag, isomer separation→ 2 peaks	SE	+NH_4, sterol frag, alkyl-pattern
TG	+NH_4, M-Q frag, alkyl-pattern	ST	+H-H_2O, alkyl-pattern

Table S3 Measurement accuracy summary compared against SRM 1950 – Metabolites in Frozen Human. Results of the fractionated human plasma

Lipid Species	Measurement* [nmol/mL]			Consensus Value** [nmol/mL]			No. of labs	Notes
DG 36:2	3.6	±	0.3	6.2	±	2.2	16	
DG 36:3	4.8	±	1.2	8.4	±	3.3	15	
TG 48:0	5.3	±	0.4	4.5	±	1.2	10	
TG 48:1	17.8	±	0.2	13	±	3.2	16	
TG 48:2	15.8	±	0.2	16	±	2.8	15	
TG 49:1	2.0	±	0.0	2.0	±	0.42	9	
TG 50:0	2.3	±	0.1	3.8	±	0.83	11	
TG 50:2	68.1	±	3.8	47	±	12	15	
TG 50:4	10.9	±	1.1	8.7	±	2.9	15	
TG 51:2	4.7	±	0.2	4.8	±	1.1	8	
TG 51:3	3.8	±	0.5	4.8	±	1.9	5	
TG 52:6	3.1	±	0.4	4.0	±	1.4	8	
TG 53:2	2.4	±	0.5	1.9	±	0.41	9	
TG 53:3	3.7	±	0.4	3.7	±	1.1	6	
TG 53:4	2.1	±	0.1	2.4	±	0.76	6	
TG 54:3	40.7	±	2.8	26	±	9.8	15	
TG 54:4	51.0	±	6.0	36	±	13	15	
TG 54:5	35.4	±	4.5	27	±	11	15	
TG 54:6	19.6	±	2.2	14	±	5.1	16	
TG 54:7	5.0	±	0.3	5.6	±	1.5	7	
TG 56:7	8.0	±	1.7	13	±	2.7	8	
LPE 18:0	1.0	±	0.0	1.6	±	0.55	15	
LPE 18:1	1.1	±	0.0	1.4	±	0.47	14	
LPE 18:2	2.3	±	0.1	1.9	±	0.56	16	
PC 32:0	10.3	±	0.8	7.2	±	1.0	18	
PC 32:1	14.7	±	1.2	13	±	1.9	18	
PC 34:1	153.0	±	3.1	120	±	21	19	
PC P-35:1/34:2	382.0	±	1.8	240	±	47	18	Includes only PC 34:2 results.
PC P-35:2/34:3	24.8	±	2.7	12	±	1.7	18	Includes only PC 34:3 results.
PC 36:1	20.3	±	1.4	26	±	4.6	17	
PC 36:2	181.5	±	6.2	140	±	25	18	
PC O-36:2/P-36:1/35:2	9.2	±	0.1	7.4	±	1.7	17	Includes only PC 35:2 results.
PC 36:3	82.7	±	4.4	100	±	14	17	
PC 36:4	173.7	±	1.2	150	±	28	19	
PC O-36:4/P-36:3/35:4	10.4	±	1.7	12	±	1.4	17	Includes only PC O-36:4 results.
PC 36:5	30.1	±	1.9	11	±	1.8	16	
PC O-36:5/P-36:4/35:5	11.2	±	0.6	6.9	±	1.6	11	Includes only PC O-36:5 results.
PC 38:3	23.8	±	2.0	26	±	5.2	14	
PC 38:4	88.9	±	4.1	84	±	14	18	
PC 38:5	39.9	±	1.9	42	±	7.9	18	
PC O-38:5/P-38:4/37:5	10.0	±	0.5	11	±	1.6	16	Includes only PC O-38:5 results.
PC 38:6	40.7	±	2.2	41	±	4.4	18	
PC O-38:6/P-38:5/37:6	5.5	±	0.7	3.6	±	1.0	12	Includes only PC O-38:6 results.
PC 40:5	7.6	±	0.3	6.7	±	1.1	18	
PC 40:6	12.7	±	1.3	14	±	2.6	17	
PE 34:1	1.3	±	0.1	1.2	±	0.17	14	
PE 34:2	2.4	±	0.0	2.2	±	0.26	16	
PE O-34:3/P-34:2	2.7	±	0.4	1.5	±	0.41	11	Includes only PE O-34:3 results.
PE 36:1	7.6	±	0.4	1.3	±	0.26	14	
PE 36:2	19.9	±	0.1	6.7	±	0.79	16	
PE 36:3	3.3	±	0.1	2.4	±	0.38	16	
PE O-36:3/P-36:2/35:3	7.9	±	0.1	3.2	±	0.76	15	Includes only PE O-36:3 results.
PE 36:4	4.1	±	0.5	3.1	±	0.39	16	
PE O-36:4/P-36:3	2.0	±	0.1	1.6	±	0.29	14	Includes only PE O-36:4 results.

Lipid Species	Measurement* [nmol/mL]			Consensus Value** [nmol/mL]			No. of labs	Notes
PE O-36:5/P-36:4	6.3	±	0.3	4.9	±	1.9	15	Includes only PE O-36:5 results.
PE 38:3	2.1	±	0.2	0.95	±	0.20	14	
PE 38:4	7.8	±	0.4	8.1	±	1.2	16	
PE O-38:4/P-38:3/37:4	1.2	±	0.0	0.94	±	0.18	9	Includes only PE O-38:4 results.
PE 38:5	1.1	±	0.1	2.7	±	0.47	12	
PE O-38:5/P-38:4	8.6	±	0.6	5.8	±	1.9	17	Includes only PE O-38:5 results.
PE 38:6	1.2	±	0.2	3.2	±	0.59	15	
PE O-38:6/P-38:5	1.1	±	0.2	4.9	±	1.2	16	Includes only PE O-38:6 results.
PE O-40:6/P-40:5/39:6	0.8	±	0.0	1.3	±	0.31	14	Includes only PE O-40:6 results.
SM d32:1	6.8	±	0.7	8.4	±	1.4	14	
SM d33:1	3.6	±	0.1	4.7	±	0.64	14	
SM d34:1	93.6	±	3.8	100	±	15	21	
SM d34:2	14.1	±	0.2	16	±	2.2	17	
SM d36:1	14.5	±	0.5	20	±	3.7	22	
SM d36:2	8.5	±	0.2	9.6	±	1.5	22	
SM d38:1	8.4	±	0.8	11	±	3.1	17	
SM d38:2	3.1	±	0.2	5.2	±	1.3	17	
SM d39:1	2.7	±	0.0	3.6	±	1.0	14	
SM d40:1	17.6	±	0.1	20	±	5.1	17	
SM d40:2	14.3	±	0.9	12	±	2.8	15	
SM d41:1	6.9	±	0.1	7.7	±	2.1	14	
SM d41:2	5.0	±	0.1	5.8	±	1.4	14	
SM d42:1	10.7	±	0.3	20	±	5.4	21	
SM d42:2	31.8	±	0.3	44	±	11	18	
SM d42:3	16.4	±	0.6	17	±	4.7	12	

Table S4 Measurement accuracy summary compared against SRM 1950 – Metabolites in Frozen Human. Results of the non-fractionated human plasma

Lipid Species	Measurement* [nmol/mL]			Consensus Value** [nmol/mL]			No. of labs	Notes
DG 36:2	13.7	±	0.9	6.2	±	2.2	16	
DG 36:3	18.7	±	0.4	8.4	±	3.3	15	
TG 48:0	6.3	±	0.0	4.5	±	1.2	10	
TG 48:1	19.3	±	0.2	13	±	3.2	16	
TG 48:2	16.4	±	0.3	16	±	2.8	15	
TG 49:1	2.0	±	0.1	2.0	±	0.42	9	
TG 50:0	3.3	±	0.7	3.8	±	0.83	11	
TG 50:2	78.1	±	2.5	47	±	12	15	
TG 50:4	12.4	±	1.6	8.7	±	2.9	15	
TG 51:2	5.6	±	0.4	4.8	±	1.1	8	
TG 51:3	4.5	±	0.8	4.8	±	1.9	5	
TG 52:6	3.8	±	1.2	4.0	±	1.4	8	
TG 53:2	2.8	±	0.4	1.9	±	0.41	9	
TG 53:3	4.6	±	0.6	3.7	±	1.1	6	
TG 53:4	2.7	±	0.4	2.4	±	0.76	6	
TG 54:3	48.0	±	0.8	26	±	9.8	15	
TG 54:4	64.8	±	1.6	36	±	13	15	
TG 54:5	43.1	±	0.4	27	±	11	15	
TG 54:6	23.8	±	0.4	14	±	5.1	16	
TG 54:7	6.2	±	0.5	5.6	±	1.5	7	
TG 56:7	10.0	±	1.8	13	±	2.7	8	
LPE 18:0	1.5	±	0.3	1.6	±	0.55	15	
LPE 18:1	1.5	±	0.0	1.4	±	0.47	14	
LPE 18:2	2.8	±	0.5	1.9	±	0.56	16	
PC 32:0	9.5	±	0.2	7.2	±	1.0	18	
PC 32:1	15.6	±	0.5	13	±	1.9	18	
PC 34:1	157.5	±	6.4	120	±	21	19	
PC P-35:1/34:2	404.6	±	10.8	240	±	47	18	Includes only PC 34:2 results.
PC P-35:2/34:3	13.1	±	0.9	12	±	1.7	18	Includes only PC 34:3 results.
PC 36:1	19.9	±	0.0	26	±	4.6	17	
PC 36:2	189.6	±	0.9	140	±	25	18	
PC O-36:2/P-36:1/35:2	6.6	±	0.1	7.4	±	1.7	17	Includes only PC 35:2 results.
PC 36:3	91.0	±	4.3	100	±	14	17	
PC 36:4	171.2	±	5.5	150	±	28	19	
PC O-36:4/P-36:3/35:4	10.6	±	0.3	12	±	1.4	17	Includes only PC O-36:4 results.
PC 36:5	11.7	±	1.6	11	±	1.8	16	
PC O-36:5/P-36:4/35:5	8.6	±	0.7	6.9	±	1.6	11	Includes only PC O-36:5 results.
PC 38:3	24.9	±	0.7	26	±	5.2	14	
PC 38:4	91.1	±	1.9	84	±	14	18	
PC 38:5	42.6	±	1.3	42	±	7.9	18	
PC O-38:5/P-38:4/37:5	11.3	±	0.6	11	±	1.6	16	Includes only PC O-38:5 results.
PC 38:6	42.3	±	3.6	41	±	4.4	18	
PC O-38:6/P-38:5/37:6	4.2	±	0.4	3.6	±	1.0	12	Includes only PC O-38:6 results.
PC 40:5	7.1	±	0.4	6.7	±	1.1	18	
PC 40:6	14.2	±	0.7	14	±	2.6	17	
PE 34:1	1.2	±	0.1	1.2	±	0.17	14	
PE 34:2	2.1	±	0.1	2.2	±	0.26	16	
PE O-34:3/P-34:2	2.7	±	0.1	1.5	±	0.41	11	Includes only PE O-34:3 results.
PE 36:1	0.8	±	0.4	1.3	±	0.26	14	
PE 36:2	3.5	±	0.4	6.7	±	0.79	16	
PE 36:3	2.3	±	0.2	2.4	±	0.38	16	
PE O-36:3/P-36:2/35:3	6.4	±	0.9	3.2	±	0.76	15	Includes only PE O-36:3 results.
PE 36:4	3.1	±	0.3	3.1	±	0.39	16	
PE O-36:4/P-36:3	1.8	±	0.2	1.6	±	0.29	14	Includes only PE O-36:4 results.

Lipid Species	Measurement* [nmol/mL]			Consensus Value** [nmol/mL]			No. of labs	Notes
PE O-36:5/P-36:4	4.3	±	0.2	4.9	±	1.9	15	Includes only PE O-36:5 results.
PE 38:3	0.4	±	0.1	0.95	±	0.20	14	
PE 38:4	2.7	±	0.4	8.1	±	1.2	16	
PE O-38:4/P-38:3/37:4	1.1	±	0.0	0.94	±	0.18	9	Includes only PE O-38:4 results.
PE 38:5	0.7	±	0.0	2.7	±	0.47	12	
PE O-38:5/P-38:4	6.3	±	1.4	5.8	±	1.9	17	Includes only PE O-38:5 results.
PE 38:6	0.5	±	0.1	3.2	±	0.59	15	
PE O-38:6/P-38:5	0.8	±	0.1	4.9	±	1.2	16	Includes only PE O-38:6 results.
PE O-40:6/P-40:5/39:6	0.5	±	0.0	1.3	±	0.31	14	Includes only PE O-40:6 results.
SM d32:1	5.5	±	0.0	8.4	±	1.4	14	
SM d33:1	3.2	±	0.0	4.7	±	0.64	14	
SM d34:1	81.2	±	0.0	100	±	15	21	
SM d34:2	11.5	±	0.0	16	±	2.2	17	
SM d36:1	15.9	±	0.0	20	±	3.7	22	
SM d36:2	9.0	±	0.0	9.6	±	1.5	22	
SM d38:1	9.9	±	0.0	11	±	3.1	17	
SM d38:2	4.3	±	0.0	5.2	±	1.3	17	
SM d39:1	3.2	±	0.0	3.6	±	1.0	14	
SM d40:1	23.6	±	0.0	20	±	5.1	17	
SM d40:2	17.0	±	0.0	12	±	2.8	15	
SM d41:1	9.8	±	0.0	7.7	±	2.1	14	
SM d41:2	7.1	±	0.0	5.8	±	1.4	14	
SM d42:1	15.9	±	0.0	20	±	5.4	21	
SM d42:2	42.9	±	0.0	44	±	11	18	
SM d42:3	22.4	±	0.0	17	±	4.7	12	

References

1. Neubauer S, Haberhauer-Troyer C, Klavins K, Russmayer H, Steiger MG, Gasser B, Sauer M, Mattanovich D, Hann S, Koellensperger G. U13C cell extract of Pichia pastoris - A powerful tool for evaluation of sample preparation in metabolomics. *J. Sep. Sci.* 2012;*35*:3091–3105.

2. Folch J, Lees M, Sloane Stanley GH. A simple method for the isolation and purification of total lipides from animal tissues. *J. Biol. Chem.* 1957;*226*:497–509.
